# 3D-cultured human placenta-derived mesenchymal stem cell spheroids enhance ovary function by inducing folliculogenesis

**DOI:** 10.1038/s41598-018-33575-9

**Published:** 2018-10-17

**Authors:** Tae-Hee Kim, Jong Ho Choi, Yesl Jun, Seung Mook Lim, Sohae Park, Jin-Young Paek, Sang-Hoon Lee, Ji-Young Hwang, Gi Jin Kim

**Affiliations:** 1 Department of Obstetrics and Gynecology, Soonchunhyang University College of Medicine Hospital, 170 Jomaru-ro, Wonmi-gu, Bucheon-si, Gyunggi-do Republic of Korea; 2 Department of Biomedical Science, CHA University, 689, Sampyeong-dong, Bundang-gu, Seongnam-si, Gyunggi-do Republic of Korea; 3 KU-KIST Graduate School of Converging Science and Technology, Korea University, 145 Anam-ro, Seongbuk-gu, Seoul Republic of Korea; 4 Department of Clinical Pathology, CHA Gangnam Medical Center, CHA University, School of Medicine, 566 Nonhyun-ro, Gangnam-gu, Seoul Republic of Korea; 5 Department of Biomedical Engineering, College of Health Science, Korea University, 145 Anam-ro, Seongbuk-gu, Seoul Republic of Korea

## Abstract

Placenta-derived mesenchymal stem cells (PD-MSCs) have numerous advantages over other adult MSCs that make them an attractive cell source for regenerative medicine. Here, we demonstrate the therapeutic effect of PD-MSCs in ovariectomized (Ovx) rats and compare their efficacy when generated via a conventional monolayer culture system (2D, naïve) and a spheroid culture system (3D, spheroid). PD-MSC transplantation significantly increased the estradiol level in Ovx rats compared with the non-transplantation (NTx) group. In particular, the estradiol level in the Spheroid group was significantly higher than that in the Naïve group at 2 weeks. Spheroid PD-MSCs exhibited a significantly higher efficiency of engraftment onto ovarian tissues at 2 weeks. The mRNA and protein expression levels of Nanos3, Nobox, and Lhx8 were also significantly increased in the Spheroid group compared with those in the NTx group at 1 and 2 weeks. These results suggest that PD-MSC transplantation can restore ovarian function in Ovx rats by increasing estrogen production and enhancing folliculogenesis-related gene expression levels and further indicate that spheroid-cultured PD-MSCs have enhanced therapeutic potential via increased engraftment efficiency. These findings improve our understanding of stem-cell-based therapies for reproductive systems and may suggest new avenues for developing efficient therapies using 3D cultivation systems.

## Introduction

The ovaries maintain the health of the female reproductive system and ensure a woman’s quality of life by balancing her hormone-producing system. Ovarian dysfunction caused by chemotherapy or age-related menopause results in systemic complications (e.g., dementia, osteoporosis, heart disease, menopausal symptoms, and metabolic syndrome)^1^. Although herbal drugs or foods can prevent menopausal complications or reduce clinical symptoms of ovarian dysfunction, medical treatments to improve premature ovarian failure and menopause are not available. At present, hormone replacement therapy is the only recommended remedy, despite the associated risk for breast cancer. For this reason, half of post-menopausal women worldwide live without reproductive hormones, such as estrogen and progesterone^1^.

Johnson and colleagues suggested that bone marrow (BM) stem cells could be a source of germ cells capable of restoring oocyte production in mouse models in which fertility has been damaged by chemotherapy or gene defects. However, in an irradiated mouse model, bone marrow transplantation (BMT) failed to result in differentiation of the transplanted cells into oocytes or to generate any improvement in ovarian function^2^. Over the last few decades, mesenchymal stem cells (MSCs) derived from several adults tissues have come to be used in regenerative medicine because they retain the potential to differentiate into multiple lineages, including endodermal, mesodermal, and ectodermal lineages, and they possess self-renewal and immunomodulatory activity^3–5^. The clinical usefulness of BM-derived MSCs (BM-MSCs) and adipose-derived MSCs (AD-MSCs) has been limited by the donor-age dependence of their stemness and the invasive procedures required for collection^6,7^.

Placenta-derived mesenchymal stem cells (PD-MSCs), in contrast, avoid the issue of donor age, can be obtained through noninvasive procedures^8,9^, and have higher self-renewal and immunomodulatory activity than BM-MSCs and AD-MSCs^7,10^. Numerous studies have characterized PD-MSCs and explored their therapeutic effects, which include anti-fibrosis, anti-inflammation, anti-apoptosis, and paracrine effects, in degenerative diseases^11–14^. Moreover, a human PD-MSC cell line (placental expanded, or PLX) is in human clinical trials for several ischemic disorders and has shown positive effects in regenerating damaged tissues (http://www.clinicaltrials.gov)^15^. However, despite this evidence that PD-MSCs can support organ regeneration, no published report has examined whether these cells can restore ovarian function.

The surrounding microenvironment is critical for directing and ensuring the therapeutic effects of implanted MSCs^16^. Thus, researchers have sought to develop new methods to enhance the function of implanted MSCs by modulating the microenvironment. Recently, three-dimensional (3D) cell culture systems, which enable cell-cell and cell-ECM interactions that mimic *in vivo* conditions much more closely than conventional monolayer (2D) cell culture systems, have become a hot topic in the fields of stem cell biology and organ regeneration^17,18^. Several studies have demonstrated that 3D spheroid MSC culture induces upregulation of adhesion molecules and proliferation in MSCs compared to adherent culture, resulting in enhancement of the therapeutic potential of MSCs^19–21^. We previously developed a polydimethylsiloxane (PDMS)-based concave microwell array using soft lithography and mold replication technology and showed that this array could be used for successful cell docking and formation of 3D cell spheroids of a desired uniform size^22,23^. This 3D culture system has proven to be an efficient tool for expanding large numbers of stem cells with controlled sizes and shapes and allows spheroids to quickly self-assemble from cell sources without the need for additional devices or prior labor^24^. Furthermore, when these 3D cell spheroids are transplanted *in vivo*, they demonstrate longer viability, good differentiation ability and greater enhancement of target organ function^25^. Therefore, we hypothesized that well-defined PD-MSC spheroids implanted in degenerated ovaries will show stable and long-term maintenance of viability and function and help to restore ovarian function.

In this paper, we demonstrate successful spheroidal 3D culture of PD-MSCs on a hemispheric concave microwell array and analyze the ability of these spheroids to improve ovarian function when implanted into a rat model of ovarian dysfunction. To verify the beneficial effects of spheroidal culture, we compared our results with those obtained using 2D-cultured PD-MSCs. We also investigated whether transplantation of 3D spheroid-cultured PD-MSCs could influence the expression levels of genes associated with ovarian growth and/or oocyte maturation. We report for the first time that well-defined (meaning uniformity in size and shape) 3D spheroid-cultured PD-MSCs can successfully be produced on a microplatform, and their implantation into ovariectomized (Ovx) rats enhances restoration of ovarian function more than implantation of isolated cells.

## Results

**Figure 1:**
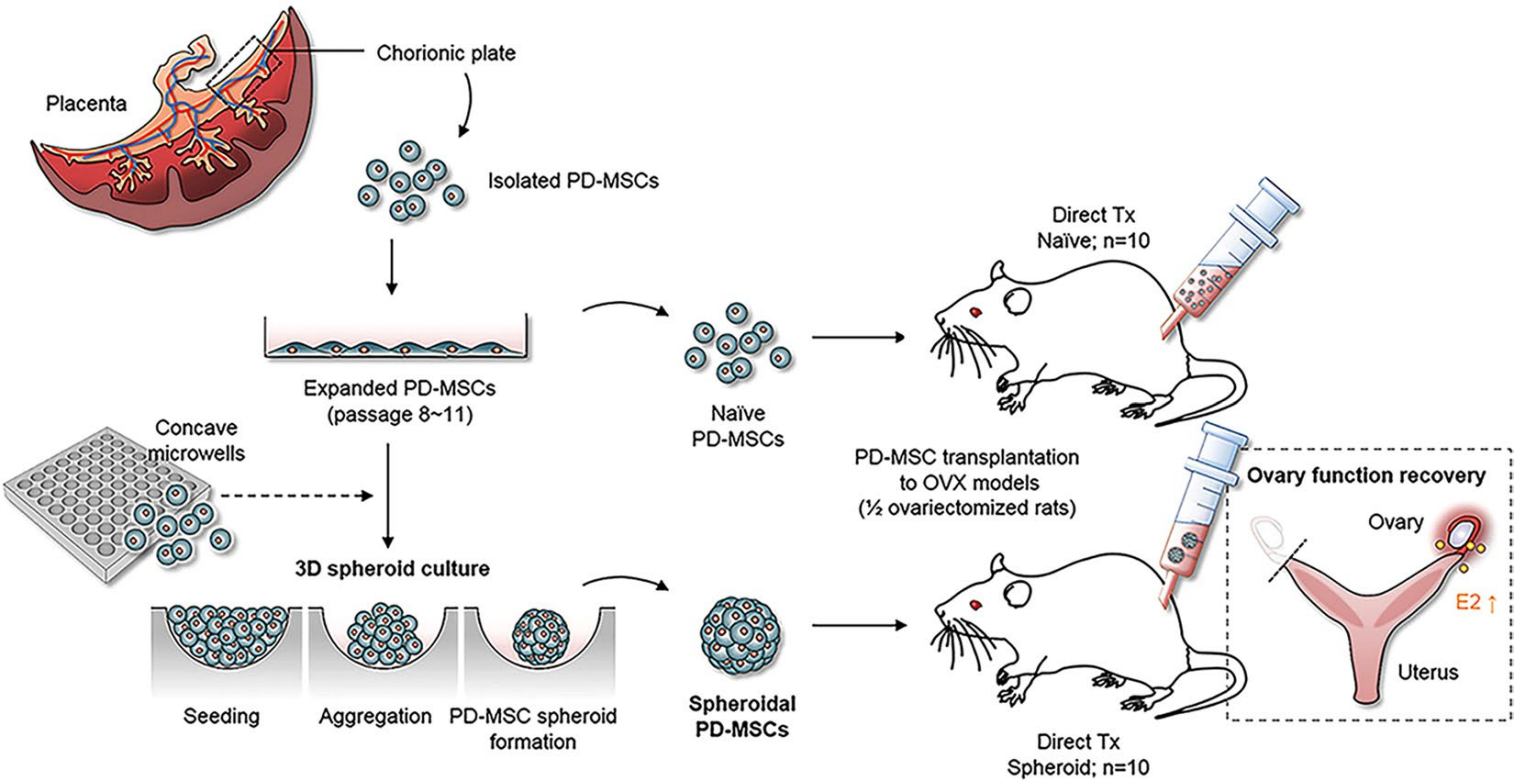
Schematic illustrations of the 3D spheroid culture of PD-MSCs and the models used to test their effect on ovary function. PD-MSCs isolated from the chorionic plates of human placenta were seeded onto PDMS-based concave microwell arrays for generation of 3D spheroids. For transplantation into ovariectomized rats, spheroidal PD-MSCs and naïve PD-MSCs were directly injected into the remaining ovary. The therapeutic effects of PD-MSCs were assessed by analysis of E_2_ production and the expression levels of folliculogenesis-related genes.

PD-MSC formation into 3D spheroids and their transplantation into Ovx rats are illustrated in Fig. 1. PD-MSCs were isolated from the chorionic plates of human placenta, subjected to both classical 2D culture and 3D spheroid-forming culture in concave microwells, and transplanted directly into our rat model. Then, we evaluated the ovarian function of rat models transplanted with 3D spheroidal structures (Spheroid group), or naïve single cells (Naïve group) and compared the results with the non-transplantation (NTx) group.

### Characterization of PD-MSCs

We previously reported the characterization of PD-MSCs^11^. Briefly, MSCs are morphologically characterized by a small cell body with a few processes that are long and thin in shape. PD-MSCs had a spindle shape similar to that of traditional MSCs (Supplementary Fig. 1a). Reverse transcription polymerase chain reaction (RT-PCR) analysis confirmed that PD-MSCs expressed self-renewal and pluripotent stem cell markers [octamer-binding transcription factor 4 (Oct-4), Nanog, SRY-related HMG-box 2 (Sox2), and telomerase reverse transcription (TERT)], as well as three germinal layer cell markers (NF68, cardiac muscle, and α-fetoprotein) and HLA-G (which has immunomodulatory effects) (Supplementary Fig. 1b). Fluorescence-activated cell sorting (FACS)-based cell cycle analysis revealed that the S and M phase PD-MSC populations constituted 15.7% and 25.3%, respectively, of the total PD-MSC population (Supplementary Fig. 1c). FACS analysis of surface markers showed that PD-MSCs were positive for mesenchymal stem cell markers (CD13, CD44, CD71, CD90, and CD105), negative for hematopoietic cell markers (CD31, CD33, CD34, and CD45), positive for human leukocyte antigen receptor (HLA)-ABC (MHC class I), and negative for HLA-DR (MHC class II) (Supplementary Fig. 1d).

To confirm the differentiation potential of PD-MSCs, we induced differentiation into various mesodermal lineages (adipogenic, osteogenic and chondrogenic). As shown in Fig. S1e, cells subjected to adipogenic differentiation showed lipid droplet accumulation upon Oil Red O staining, and those subjected to osteogenic differentiation showed an increase in the level of densely mineralized phosphate deposits upon von Kossa staining. Chondrogenic differentiation ability was detected in PD-MSCs with Alcian blue staining. Also, teratoma formation in NOD/SCID mice after PD-MSCs transplantation was not detected (Supplementary Fig. 2). These results suggest that the PD-MSCs have the potential for self-renewal and differentiation into mesodermal lineages.

### Formation of PD-MSC spheroids in concave microwells

**Figure 2:**
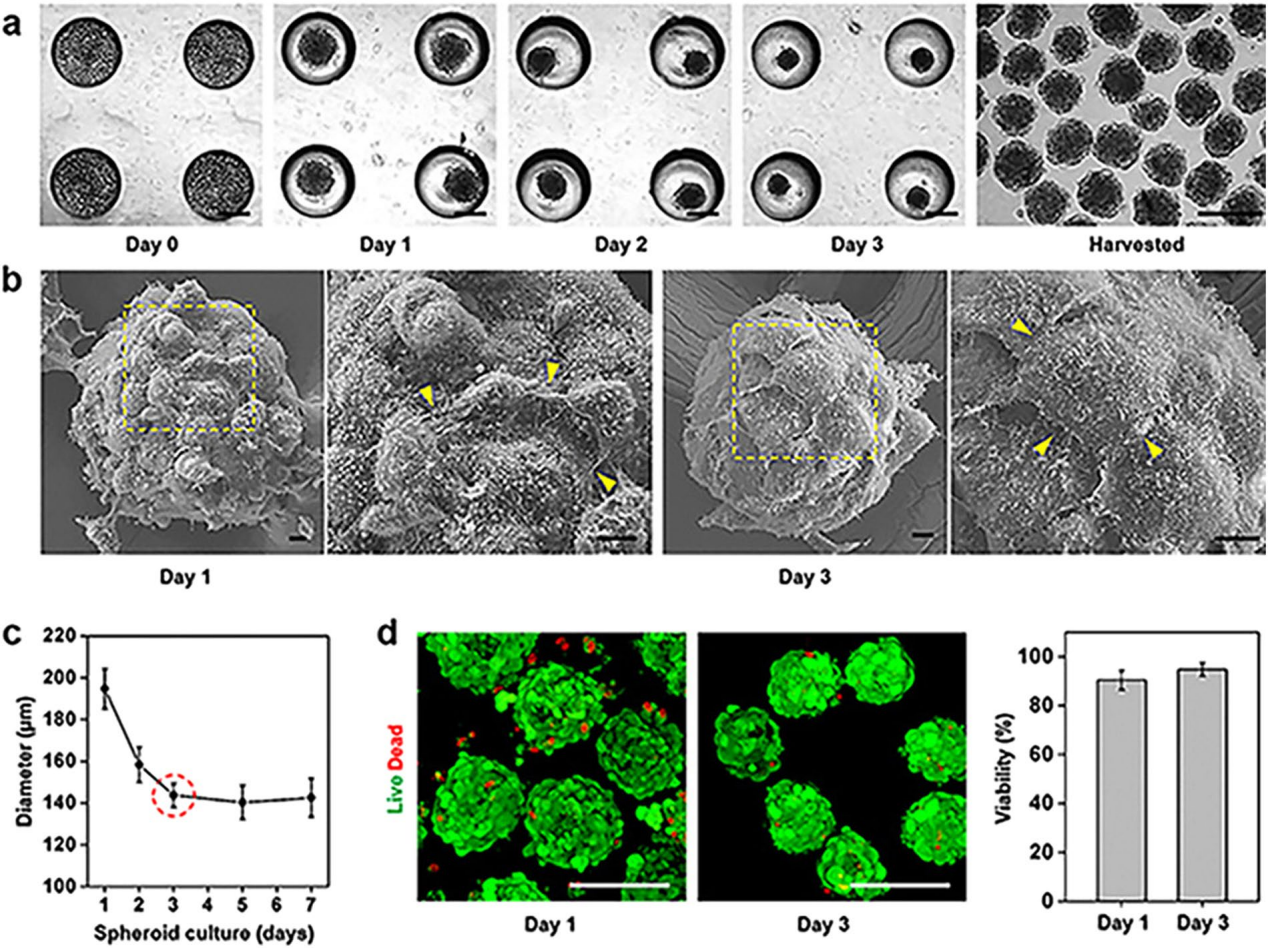
Morphologies and viabilities of PD-MSC spheroids. (**a**) Optical images of PD-MSC spheroids forming in concave microwells over time. Scale bars: 200 µm. (**b**) SEM images of spheroids on days 1 (left) and 3 (right) with magnified surface images showing differences in the junctions between neighboring cells (arrowheads). Scale bars: 10 nm. (**c**) Analysis of the diameter of PD-MSC spheroids over time. (**d**) Cell viabilities in spheroids cultured for 1 and 3 days. Scale bars: 200 µm. The quantification data (right) are expressed as the mean ± SE (n = 25).

Naïve PD-MSCs were cultured in PDMS-based concave microwells, and their morphologies were assessed over time (Fig. 2a). Seeded PD-MSCs were maintained in the undifferentiated state by culture in optimized medium supplemented with FGF-4 and heparin (Supplementary Fig. 3). The PD-MSCs started to aggregate immediately after seeding, formed spheroidal structures on day 1, and presented as compact, smooth-surfaced spheroids after day 2. When harvested for transplantation, the spheroids appeared monodisperse, and their size was perfectly regulated by the diameter of the concave microwells. As shown in Fig. 2b, scanning electron microscopy (SEM) imaging revealed that the spheroids appeared to have rough surfaces with rounded cells on day 1, but exhibited smooth and even surfaces with tightly connected, flat outer cells on day 3. Magnified SEM images showed that the shapes of the cells that formed cell-cell junctions differed on days 1 and 3. PD-MSC spheroids cultured for 1 day in 500-μm-diameter microwells had an average size of 194.7 ± 9.6 μm (Fig. 2c). The average size decreased to 143.7 ± 5.7 μm on day 3 and thereafter remained relatively consistent through days 5–7. In Fig. 2d, the green and red fluorescent signals indicate live and dead cells, respectively, in PD-MSC spheroids on days 1 and 3. Interestingly, the cellular debris on the spheroid surface disappeared over time. Quantification showed that the cells within the spheroids were highly viable on days 1 and 3 (90.4 ± 3.9% and 94.8 ± 2.7%, respectively), suggesting that the PDMS mold did not affect PD-MSC viability during cultivation.

### PD-MSCs restore estradiol production in ovariectomized rats

**Figure 3:**
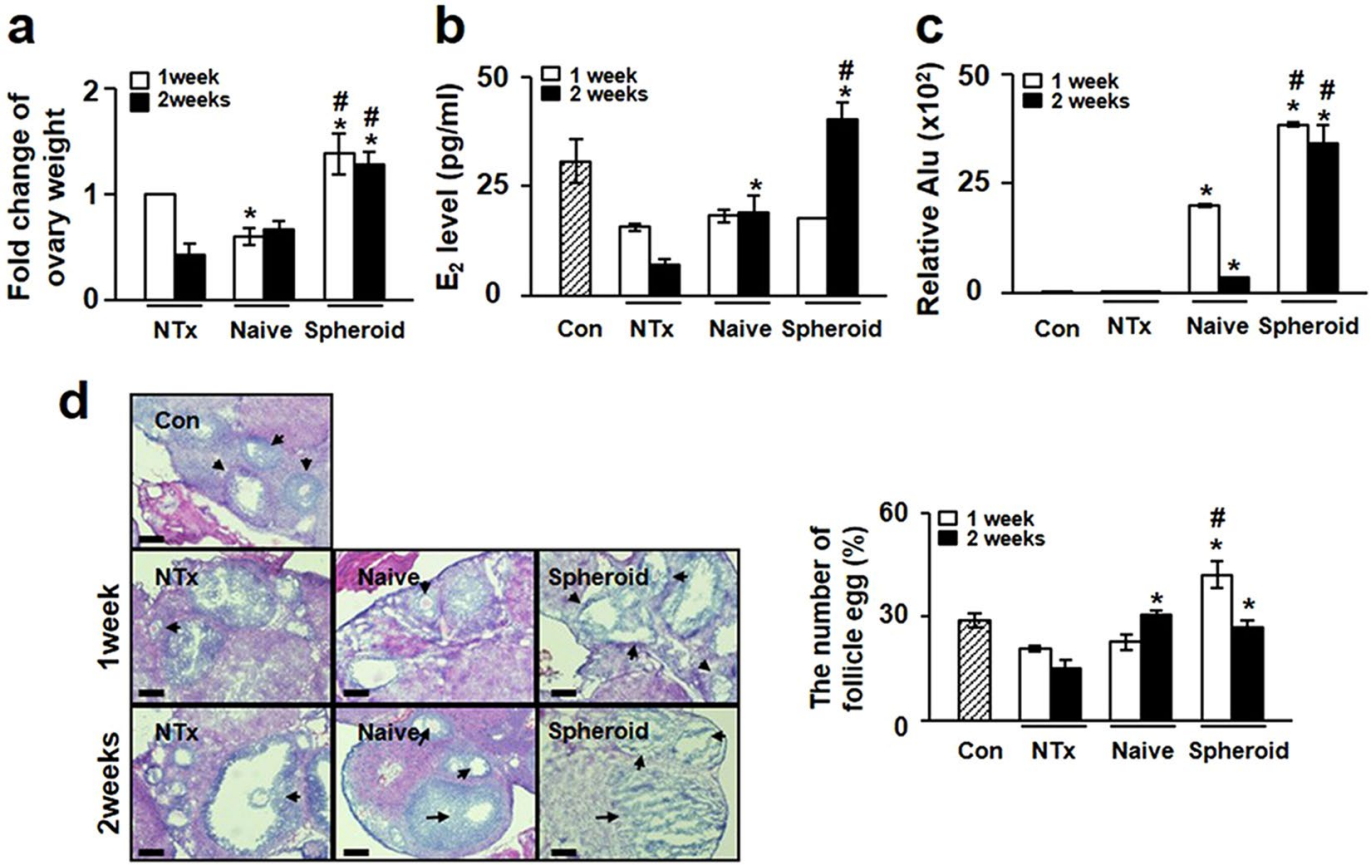
Therapeutic effect of PD-MSCs in ovariectomized (Ovx) rats. (**a**) The graph represents the ratio of ovary weight to body weight in Ovx rats transplanted with naïve or spheroid PD-MSCs. (**b**) E_2_ levels in plasma samples from transplanted and non-transplanted rats. (**c**) Quantification of the expression of human-specific Alu sequences in rat ovary tissues engrafted with naïve PD-MSCs or spheroidal PD-MSCs, as assessed by qRT-PCR. (**d**) Histological analysis of ovary tissues obtained from Ovx rats transplanted with or without naïve or spheroidal PD-MSCs (left). Quantification of follicles in rat ovary tissues according to PD-MSC transplantation (right). The arrows indicate follicles below 100 µm in size. Scale bar: 20 nm. The data are expressed as the mean ± SE. Symbols: ^*^represent a significant difference compared to the NTx group (*p* < *0*.*05*); ^#^represents a significant difference between the Naïve and Spheroid groups (*p* < *0*.*05*). Abbreviations: Ovx, ovariectomized (unilateral); Con, sham-surgery group; NTx, non-transplanted Ovx model; Naïve, naïve PD-MSC-transplanted group; and Spheroid, spheroid PD-MSC-transplanted group.

To evaluate the effect of PD-MSCs in the Ovx rat model, we first analyzed the ratio of ovary weight to total body weight. In the NTx group, the ratio decreased in the weeks following ovariectomy; in contrast, the ratios in the Naïve and Spheroid groups remained fairly constant. Interestingly, the ratios in the Spheroid group were significantly higher than those in the NTx and Naïve groups at 1 and 2 weeks post-surgery (*p* < *0*.*05*) (Fig. 3a).

Estrogen is a major ovary-secreted hormone, and the level of estradiol (E_2_) in the blood can be a marker of ovarian function. Here, we examined serum E_2_ levels to determine whether PD-MSC transplantation affected ovarian function in our rat model. As shown in Fig. 3b, the level of serum E_2_ in the NTx group was decreased by ~50% compared to the normal control (non-Ovx) group (30 ± 4.8 pg/ml *vs*. 15.58 ± 0.76 pg/ml; *p* < *0*.*05*) at 1 week and by 77% of the control level (30 ± 4.8 pg/ml *vs*. 6.9 ± 1.4 pg/ml; *p* < *0*.*05*) at 2 weeks. The rats with transplanted PD-MSCs had higher E_2_ levels (Naïve group, 18.9 ± 3.8 pg/ml; Spheroid group, 40.3 ± 3.8) compared with the NTx group at 2 weeks (*p* < *0*.*05*). Furthermore, the E_2_ level in the Spheroid group was significantly higher than that in the Naïve group at 2 weeks (*p* < *0*.*05*) (Fig. 3b). High stem cell engraftment efficiency is well known to maximize therapeutic outcomes. To confirm that the human PD-MSCs were transplanted onto rat ovarian tissue, we investigated the expression of human-specific Alu sequences using real-time PCR. These sequences were absent from the control and NTx groups but were present in the Naïve and Spheroid groups. Human Alu sequence expression levels decreased over time in the latter two groups, but the expression of Alu sequences in the Spheroid group was consistently higher than that in the Naïve group at all tested time points (Fig. 3c). These results indicate that the PD-MSC spheroids showed an increased efficiency of engraftment onto rat ovarian tissues. This is likely to prolong their half-life in ovarian tissues and accounts for the increase in the circulating E_2_ level. To examine whether PD-MSC transplantation can increase the number of follicles in the utilized rat model, we also determined the total number of follicles in the collected ovaries. As shown in Fig. 3d, the number of follicles was significantly lower in Ovx rats compared with the sham-surgery control group (*p* < *0*.*05*), and PD-MSC transplantation enhanced the number of follicles compared with the NTx group at 2 weeks (30.3 ± 1.2 *vs*. 15 ± 2.5, *p* < *0*.*05*). Interestingly, the number of follicles in the ovary was dramatically increased in the Spheroid group at 1 week (*p* < *0*.*05*, Fig. 3d). These results suggest that PD-MSC transplantation into the ovary can restore ovarian function in Ovx rats in a short period. This indicates that spheroid PD-MSCs had a higher therapeutic effect than naïve PD-MSCs in the utilized model *via* the increased E_2_ level.

### Transplantation of spheroid PD-MSCs promotes folliculogenesis-related gene expression in ovariectomized rats

**Figure 4:**
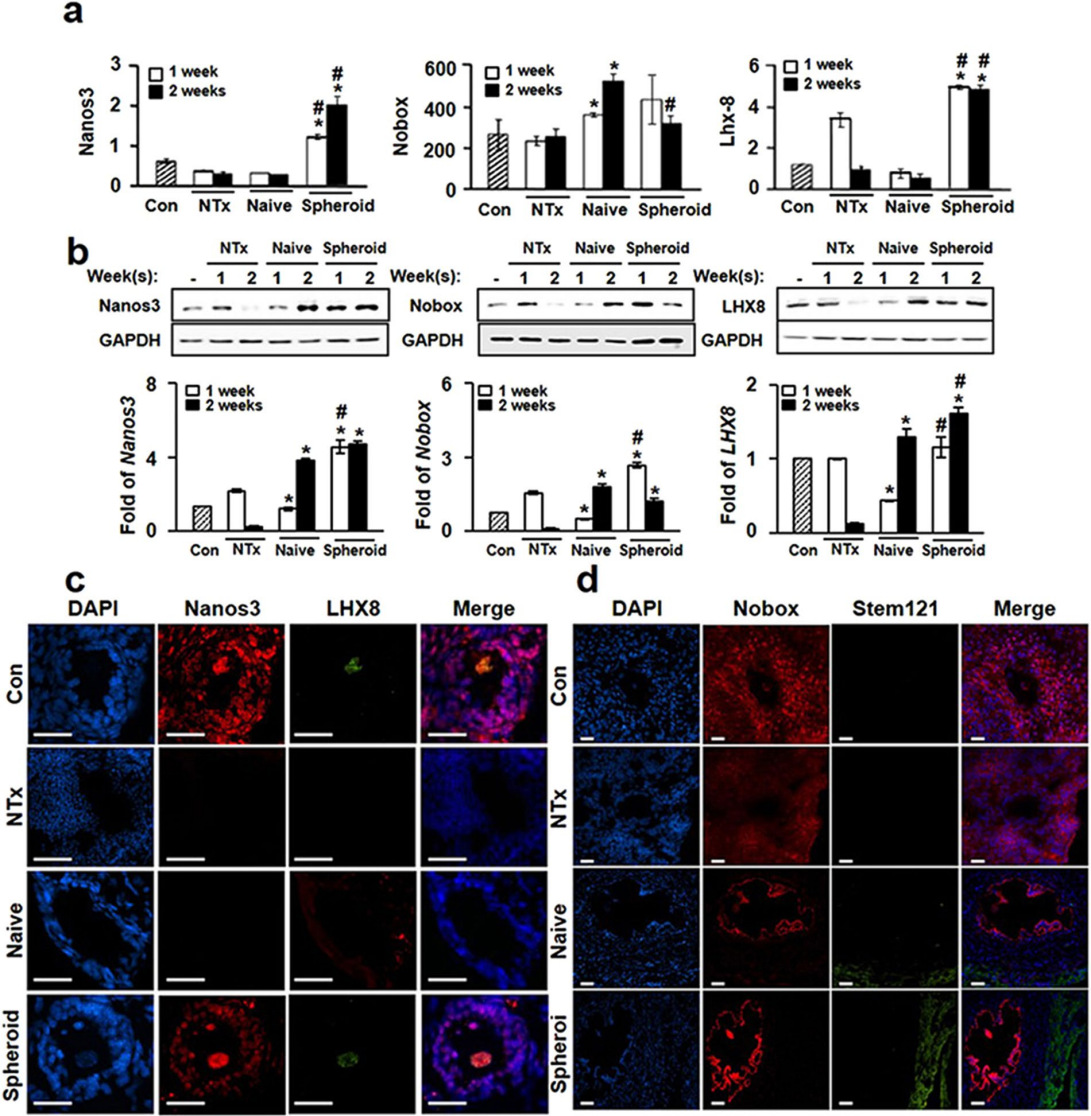
Analysis of oogenesis-related gene expression in ovary tissues of Ovx rats with or without PD-MSC transplantation. (**a**) The mRNA expression levels of Nanos3 (left), Nobox (middle) and Lhx8 (right) were assessed with qRT-PCR. (**b**) The protein expression levels of Nanos3, Nobox and Lhx8 were assessed with Western blot analysis. (**c**) Localization of Nanos3 and Lhx8 in rat ovary tissues, as assessed by double immunofluorescence (red  LNanos3; green = Lhx8; blue = nuclei, DAPI). Scale bar: 20 nm (x200 original magnification). (**d**) Localization of Nobox and stem121 in rat ovary tissues, as assessed with double immunofluorescence (red = Nobox; green  gstem121; blue tenuclei, DAPI). Scale bar: 20 µm (x100 original magnification). The data are expressed as the mean ± SE. Symbols: ^*^represents a significant difference compared to the NTx group (*p* < *0*.*05*); and ^#^represents a significant difference between the Naïve and Spheroid groups (*p* < *0*.*05*). Abbreviations: Ovx, ovariectomized (unilateral); Con, sham-surgery group; NTx, non-transplanted Ovx model; Naïve, naïve PD-MSC-transplanted group; and Spheroid, spheroid PD-MSC-transplanted group.

Normal folliculogenesis, which is required for proper maturation and function of the ovary, is affected by various factors, including the environment and the expression of major folliculogenesis-related genes, such as Nanos3, Nobox (newborn ovary homeobox) and Lhx8 (LIM-homeobox protein 8). Accordingly, we examined whether PD-MSC transplantation triggered the expression of these key genes in the utilized rat model of ovary dysfunction. qRT-PCR analysis revealed that the mRNA expression levels of Nanos3, Nobox, and Lhx8 in the Spheroid group were significantly higher than those in the NTx group at 1 and 2 weeks, and expression levels were higher than those in the normal control group (*p* < *0*.*05*) (Fig. 4a). Consistent with this, the protein expression levels of these factors were higher in the Spheroid group relative to the NTx group at 1 and 2 weeks (*p* < *0*.*05*). Furthermore, Lhx8 expression in the Spheroid group was significantly higher than that in the Naïve group at all weeks (*p* < *0*.*05*) (Fig. 4b). These results indicate that PD-MSC spheroid transplantation effectively enhanced the expression of folliculogenesis-related genes in Ovx rats compared to the Naïve group. Finally, we used double immunofluorescence to confirm whether folliculogenesis-related gene expression was increased by PD-MSCs or co-localized with the engrafted PD-MSCs. In the normal ovary, Nanos3 and Nobox were detected in the granules and oocytes, while Lhx8 was detected only in the oocytes (Fig. 4c). However, expression of these genes was absent in the follicles of the NTx group. In the transplanted groups, Nanos3 and Nobox were detected in the granules of Naïve rats, while Lhx8 was detected in oocytes of the Spheroid group (Fig. 4c). In addition, the expression of stem121 (a human-specific protein) was high in the ovary tissues of the Spheroid group compared to the Naïve group (Fig. 4d). These results suggest that PD-MSC transplantation upregulated the expression of folliculogenesis-related genes in our rat model, and the therapeutic effects of spheroid PD-MSCs were higher than those of naïve PD-MSCs.

## Discussion

Premature ovarian failure (POF) and early menopause result in hormone deficiencies that increase the rate of mortality, coronary heart disease, dementia, osteoporosis, mood changes (e.g., senile depression), sexual dysfunction, and atrophic vaginitis^1^. Despite these serious consequences, the best available treatment for POF is hormone replacement therapy, which carries a risk for thromboembolism, stroke, vaginal bleeding, heart disease, and breast cancer^26,27^. Recently, stem cell therapies have been used clinically to treat certain degenerative diseases (e.g., urinary incontinence, type 1 diabetes mellitus, cardiomyopathy, and complications of Crohn’s disease), to regenerate bone or cartilage, and to provide immune support during chemotherapy^28,29^. Thus, we speculated that stem cells might restore the function of damaged ovaries. However, relatively few studies have examined the use of stem cell therapy to treat ovarian dysfunction.

In previous studies, the application of BM-MSCs, AD-MSCs and umbilical cord blood stem cells in rat models of ovarian dysfunction have been reported, and transplantation of these cells had positive effects in the damaged ovary tissues, such as an increased E_2_ level and anti-inflammatory effects^28,30^. Additionally, Lai *et al*. demonstrated that skin-derived and endometrial MSCs helped restore ovarian function in a POF mouse model by downregulating pro-inflammatory cytokines^31,32^ and Wang *et al*. reported that granulosa cells differentiated from human amniotic epithelial cells could restore folliculogenesis in a mouse model of chemotherapy-induced POF^33^. However, the therapeutic mechanisms and optimal transplantation conditions remain unclear. In the present study, we show for the first time that PD-MSC transplantation can significanlty restore ovarian function by altering the expression levels of folliculogenesis-related genes, E_2_ production, and the number of follicles according to the form of the transplanted PD-MSCs. Furthermore, we present novel results showing that transplantation of 3D-cultured spheroidal PD-MSCs yields increased engraftment onto ovary tissues, and the efficiently engrafted cells induce a significant increase in estrogen production, which may yield enhanced therapeutic effects in Ovx rats.

During mammalian oogenesis, ovarian follicular renewal activity determines the number and quality of the new oocytes and primordial follicles through interaction between germ line cells and ovarian follicles^34^. Abnormalities in growth and maturation of follicles (such as those triggered by chemicals or developmental problems) can lead to POF or menopause. In rodent models, chemicals (e.g., busulfan and cyclophosphamide, B/C) that destroy primordial oocytes can trigger reversible POF^35^, while surgical ovariectomy can create an irreversible model of ovarian dysfunction. Here, we investigated the effect of PD-MSCs on folliculogenesis in Ovx rats and show for the first time that these cells can restore ovarian function, at least in part, by regulating the ovarian follicle microenvironment to induce gene expression. We also further show that the use of 3D spheroid-cultured PD-MSCs significantly increases the number of ovarian follicles and the expression levels of folliculogenesis-related genes in a short period compared to 2D-cultured (naïve) PD-MSCs.

Spheroid culture techniques, such as hanging drop and spinner flask methods, have a limited ability to produce 3D spheroids of uniform size and shape and may suffer from size-dependent issues associated with central hypoxia^36^. In our system, concave microwells facilitate cell aggregation and generate spheroids with a uniform size and shape. The concave curvature also minimizes the contact of microwells with the spheroids, making them easy to harvest without additional processing. This method may therefore help improve the efficacy of MSC-based therapeutics. Microarray analyses have revealed drastic differences in the gene expression profiles of MSCs grown in 2D monolayers and 3D spheroid cultures, with reported upregulation of 1,731 genes and downregulation of 1,387 genes^37^. In previous studies, spheroid formation was shown to enhance the efficiency of biological processing to more closely mimic that seen in an *in vivo* system^19,38–40^. For example, Santos *et al*. reported that the environment surrounding Wharton’s jelly-derived MSCs (WJ-MSCs) in umbilical cord tissue spheroids mimicked the native cell microenvironment, yielding a richer secretome profile compared with 2D-cultured WJ-MSCs. Moreover, the same authors demonstrated that the 3D-conditioned medium of such cells could enhance wound healing *in vivo*^41^. Additionally, Lee *et al*. compared the therapeutic efficacy of umbilical cord blood MSC spheroids compared with the usual MSC suspension in repairing infarcted myocardium and found that spheroid culture enhanced the therapeutic efficacy via activation of E-cadherin, which triggers ERK/AKT signaling to support the proliferative and paracrine activities of MSCs^42^. In future studies, the concave microwell arrays of our 3D spheroid culture system could be connected to a microfluidic channel, which could be used to deliver fresh media and/or signaling molecules and cytokines secreted by other cells^43,44^. Furthermore, physical cues (e.g., shear stress) could be applied to cells within spheroids to test the effect on the fate and behavior of stem cells. Going forward, we expect that well-defined spheroids such as those generated in the present work will be prepared using microfluidic platforms and tested against diverse stimuli and stressors and that such work will contribute to furthering the clinical potential of this system for regenerating ovarian function. Although compensatory hypertrophy of ovary tissues by spheroid PD-MSC transplantation should be validated in the future, our present results indicate that PD-MSC transplantation can restore ovarian function by inducing ovarian folliculogenesis. In addition, the spheroid form of PD-MSCs appears to confer enhanced therapeutic potential to PD-MSCs transplanted into Ovx rats through increased engraftment efficiency. Thus, cell therapy using PD-MSC spheroids could effectively restore ovarian function and expand the functional period of a woman’s ovaries, improving her quality of life and reducing medical costs due to age-related disease and morbidity. Therefore, our findings provide new insights into the mechanisms by which stem cell-based therapeutics act on the reproductive system and suggest new avenues for developing more efficient 3D-culture-based therapies.

## Materials and Methods

### Animals

Thirty-five eight-week-old female Sprague-Dawley rats (Orient Bio Inc., Korea) were randomly divided into groups. All procedures were performed in accordance with the ethical guidelines published by the Research Institute of Genexine Co. (Korea). Unilateral ovariectomy (Ovx) was performed as previously described^45^, with the following modifications. Briefly, rats were anesthetized by intramuscular injection of ketamine and Rompun (3:1; 100 μl/g body weight), and surgical procedures were performed. A single 3-cm incision was made to expose the lower portion of back muscles. A 1-cm incision was made in the muscles overlying the right ovary, which was isolated, tied off with a sterile suture, and harvested. The rats were allowed to recover for 1 week. All animal experimental processes employed protocols consistent with the Institutional Review Board of CHA General Hospital (Korea, IACUC-130009).

### Cell culture of PD-MSCs

The utilized human normal term placenta (38–40 weeks) had no evidence of any medical, obstetrical, or surgical complication. All participants provided written informed consent prior to placenta collection. The collection and use of placenta were approved by the Institutional Review Board of the Kangnam CHA General Hospital, Seoul, Korea (07–18). PD-MSCs were isolated as described previously^11^. Briefly, the fetal membrane was removed from the chorionic plate of each placenta, and cells were treated with 0.5% collagenase IV (Sigma-Aldrich) for 30 min at 37 °C. The harvested cells were plated (2 × 10^5^ cells/cm^2^) with Ham’s F-12/DMEM supplemented with 10% fetal bovine serum (FBS) and 1% penicillin-streptomycin (PS) (Life Technologies).

### Analysis of cell growth and cell cycle

PD-MSCs were subjected to cell cycle analysis using FACS. Cells (1 × 10^6^) were harvested, fixed in 70% ethanol, and then incubated with 0.5 μg/ml RNase and 50 μg/ml propidium iodide (Sigma) for 30 min at 37 °C. The treated cells were analyzed with a FACS Vantage SE Cell Sorter equipped with ModiFit LT software (BD Bioscience).

### Spheroid culture and viability of PD-MSCs

Spheroids were generated on PDMS-based concave microwells fabricated using soft lithography techniques and the meniscus of the PDMS prepolymer, as previously described^46^. The concave micromolds consisted of 1 × 1 cm arrays of 500-μm diameter microwells, for a density of 100 wells per cm^2^. All substrates were coated with 3% (w/v) bovine serum albumin to prevent cell attachment. Trypsin-dispersed single PD-MSCs (200,000 cells per mold, passages 8–11) were seeded on top of the concave microwells, which allowed the cells to become trapped within the wells. At 5 min post-seeding, a flow of culture medium was gently applied to remove non-docked cells. The plated cells were cultured with medium containing FGF-4 (75 ng/ml) and heparin (3 μg/ml) (Peprotech). For measuring viability, spheroids were labeled with calcein-AM and ethidium homodimer-1 (EthD-1; Molecular Probes). Confocal microscope images were used for measuring diameters of spheriods and viability, and ImageJ software was used for quantification.

### Scanning electron microscopy (SEM)

Spheroids were firstly fixed with 2.5% glutaraldehyde and secondly fixed in 1% osmium tetroxide after gentle washing, for 1 h respectively. The fixed spheroids were dehydrated with a graded ethanol series (25%, 50%, 75%, 95%, and 100%), immersed in tert-butyl alcohol (three times, 30 min each) at RT, and then frozen at −70 °C. The tert-butyl alcohol was evaporated by freeze drying, and the spheroids were mounted on specimen stubs with graphite paste, coated with palladium alloy, and observed under SEM (JEOL, Japan).

### Differentiation of PD-MSCs

To induce osteogenic differentiation, PD-MSCs were plated at 5 × 10^3^/cm^2^ in the medium containing 1 μM dexamethasone, 10 mM glycerol 2-phosphate, 50 μM L-ascorbic acid 2-phosphate, 10% FBS and 1% PS. After 21 days, the cells were subjected to von Kossa staining. To visualize accumulated calcium deposits, cells were incubated with 5% silver nitrate (Sigma) under 100 W lamp light. To induce adipogenic differentiation, PD-MSCs were seeded at 5 × 10^3^/cm^2^ in the medium containing 1 μM dexamethasone, 0.5 mM isobutyl methylxanthine, 0.2 mM indomethacin, 1.7 μM insulin, 10% FBS and 1% PS. After 21 days, the cells were fixed with 4% paraformaldehyde (PFA). For visualization of lipid vesicles, cells were incubated for 1 h with Oil Red O (Sigma), and nuclei were counterstained with Mayer’s hematoxylin (DAKO). To induce chondrogenic differentiation, cell pellets by centrifugation of 5 × 10^5^ cells at 1,000 rpm for 5 min were cultured in the medium containing 100 nM dexamethasone, 100 mM sodium pyruvate, 50 nM L-ascorbic acid-2 phosphate (all from Sigma), insulin-transferrin-selenium premix (Gibco), TGF-β-1 (Peprotech), and 1% PS. After 21 days, the cell pellets were fixed (4% formaldehyde), cryo-sectioned (10 um), and stained with Alician blue (Sigma).

### Fluorescence-activated cell sorting (FACS) analysis

Cells were stained with anti-CD31-PE, anti-CD33-FITC, anti-CD34-PE, anti-CD45-FTIC, anti-CD13-PE, anti-CD44-PE, anti-CD45-FITC, anti-CD71-PE, anti-CD90-PE, anti-CD95-APC, anti-HLA-ABC-FITC, anti-HLA-DR-FITC (all from BD Bioscience), anti-CD31-APC (eBioscience), anti-CD13-PE (Biolegend) and anti-CD105-FITC (R&D Systems). The stained cells were analyzed with a FACSCalibur flow cytometer (Becton Dickinson).

### Transplantation of PD-MSCs into ovariectomized rats

Three days-cultured PD-MSC spheroids were harvested from microwells. The estimated number of trapped cells was approximately 25,000 per mold, forming 100 spheroids per mold after cultivation. Naïve PD-MSCs and PD-MSC spheroids (100,000 cells, respectively) were stained using a PKH26 Fluorescent Cell Linker Kit (Sigma) and directly transplanted into the remaining ovary beginning one week after ovariectomy. Non-transplanted rats (NTx) were sham-transplanted with culture medium (n hr10, respectively). Blood samples were harvested weekly, and plasma was collected in EDTA-coated tubes, separated by centrifugation and stored at −80 °C until it was measured for plasma E_2_ using an Estradiol DSL-4400 Radioimmunoassay kit (Diagnostic Systems Laboratories). Rats of all groups were sacrificed at 1 and 2 weeks post-surgery, ovary tissues were obtained, and ovary weights were measured.

### Reverse transcription polymerase chain reaction (RT-PCR)

Total RNA of the cells was extracted with an RNeasy plus mini kit (Qiagen), and cDNA was synthesized from 1 μg of total RNA using Superscript III reverse transcriptase (Invitrogen). PCR amplification was performed with specific primers (Table 1). The cDNA was subjected to 35 cycles of amplification at 95 °C for 20 s and at the appropriate annealing temperature for the utilized primers for 40 s. The amplified PCR products were electrophoresed on agarose gels containing ethidium bromide, and bands were visualized under UV light.

**Table 1:** Primers used in the present study for RT-PCR analysis.

Name	Direction	Sequence (5′ → 3′)	Tm (°C)
Oct-04	Sense	AGT GAG AGG CAA CCT GGA GA	54
	Antisense	GTG AAG TGA GGG CTC CCA TA	
Nanog	Sense	TTC TTG ACT GGG ACC TTG TC	54
	Antisense	GCT TGC CTT GCT TTG AAG CA	
Sox-2	Sense	AGA ACC CCA AGA TGC ACA AC	52
	Antisense	GGG CAG CGT GTA CTT ATC CT	
NF-68	Sense	GAG TGA AAT GGC ACG ATA CCT A	58
	Antisense	TTT CCT CTC CTT CTT CTT CAC CTT C	
Cardiac	Sense	GGA GTT ATG GTG GGT ATG GGT C	58
	Antisense	AGT GGT GAC AAA GGA GTA GCC A	
AFP	Sense	ATG CTG CAA ACT GAC CAC GC	55
	Antisense	GCT TCG CTT TGC CAA TGC TT	
HLA-G	Sense	GCG GCT ACT ACA ACC AGA GC	58
	Antisense	GCA CAT GGC ACG TGT ATC TC	
β-actin	Sense	TCC TTC TGC ATC CTG TCA GCA	58
	Antisense	CAG GAG ATG GCC ACT GCC GCA	
Nanos3	Sense	CTC TGC ATG AGG AAG AGG AGC C	60
	Antisense	GGA CTG ATA GAT CGC ACG AGA	
Nobox	Sense	AGC CAG TGC AGA TCT GCA CC	60
	Antisense	TGT CAC TGC CAG GAA CAT CCC TC	
Lhx8	Sense	GTA TCA CTT GGC TTG CTT	60
	Antisense	ATT ACC GTT CTC CAC TTC	
GAPDH	Sense	GGA AAG CTG TGG CGT GAT	60
	Antisense	AAG GTG GAA GAA TGG GAG TT	

### Quantitative real-time PCR (qRT-PCR) analysis

Frozen ovaries were pooled and homogenized, and RNA was extracted. qRT-PCR was performed with 300 ng of target RNA and Alu-specific primers^47^ (Applied Biosystems) using a SYBR ExScript RT-PCR Kit (TaKaRa) and cycling conditions of 2 min at 95 °C, followed by 40 cycles of 5 s at 95 °C and 30 s at 56 °C. The primer sequences for the human Alu-sequence primers and β-actin were 5′-CTGGGCGACAGAACGAGATTCTAT-3′ (forward) and 5′-CTCACTACTTGGTGACAGGTTCA-3′ (reverse) for the Alu sequence and 5′- TCC TTC TGC ATC CTG TCA GCA-3′ (forward) and 5′- CAG GAG ATG GCC ACT GCC GCA-3′ (reverse) for β-actin. The values were normalized to rat β-actin gene (in triplicate). The oogenesis-related proteins Nanos3, Nobox and Lhx8, their mRNA levels were determined with cycling conditions of 10 min at 95 °C, and 40 cycles of 10 s at 95 °C and 30 s at 59 °C (in duplicate). The values were normalized to rat GAPDH gene (Table 1).

### Western blot analysis

Ovary tissues in each group were homogenized and lysed in protein lysis buffer (Sigma). Equal amounts of protein lysates from individual animals were pooled, resolved on 10% sodium dodecyl sulfate polyacrylamide gels, and transferred to PVDF membranes (Bio-Rad Laboratories). The membranes were blocked and incubated with primary antibodies of anti-Lhx8 (1:1,000, Santa Cruz Biotechnology), anti-Nanos3 (1:1,000, Abcam, Cambridge) and anti-Nobox (1:1,000, Abcam) and reacted with a secondary antibodies of anti-rabbit IgG (1:10,000; Bio-Rad Laboratories) or anti-goat IgG (1:5,000; Santa Cruz Biotechnology). The bands were detected using a ChemiDoc system (Bio-Rad Laboratories). All reactions were performed in triplicate for quantification.

### Histological analysis

To detect oocyte maturation, ovaries were fixed and embedded in paraffin, and serial sections (5-µm) were stained with hematoxylin and eosin solutions (Sigma). Images were detected using a Zeiss Axioskop2 MAT microscope (Carl Zeiss MicroImaging), and the total number of follicles was determined. For immunofluorescence analysis, ovary tissues were embedding in a cryomold with compound (Sakura), frozen on dry ice and stored at −80 °C. For imaging, the samples were serially sectioned (5-µm) and fixed with 100% methanol. The sections were washed, blocked for 1 h at RT using Protein Block Serum-Free buffer, and then incubated overnight at 4 °C with a 1:100 dilution of primary antibodies as follows: anti-Nanos3 (Abcam), anti-Lhx8 (Santa Cruz Biotechnology), anti-Nobox (Abcam) and anti-stem121 (StemCells, Inc.) in Antibody Diluent with Background Reducing Components (Dako). After then, the sections were incubated for 90 min at RT with 1:200 dilutions of Alexa Fluor 594-conjugated goat anti-rabbit IgG and Alexa Fluor 488-conjugated mouse anti-goat IgG (Santa Cruz Biotechnology). The sections were counterstained with 4′,6-diamidino-2-phenylindole (DAPI, Dako). The number of follicles was determined, and follicles less than 100 µm in size in each rat were quantified. All experiments were performed in triplicate.

### Statistical analysis

The data are expressed as the mean ± standard error (SE). ANOVA was used for between-group comparisons of the data obtained at various time points. A *p* value less than 0.05 was considered statistically significant.

## Electronic supplementary material

Supplementary information and data
