# Supplementary material for: 3D-cultured human placenta-derived mesenchymal stem cell spheroids enhance ovary function by inducing folliculogenesis
Source: Sci Rep. 2018 Oct 17;8:15313. doi: 10.1038/s41598-018-33575-9 (PMC6193033; doi:10.1038/s41598-018-33575-9)
Supplement: Supplementary file 1 — Supplementary information and data [file 41598_2018_33575_MOESM1_ESM.pdf]

Supplementary Information

3D-cultured human placenta-derived mesenchymal stem cell spheroids enhance ovary function by inducing folliculogenesis

Tae-Hee Kim, Jong Ho Choi, Yesl Jun, Seung Mook Lim, Sohae Park, Jin-Young Paek, Sang-Hoon Lee, Ji-Young Hwang, Gi Jin Kim

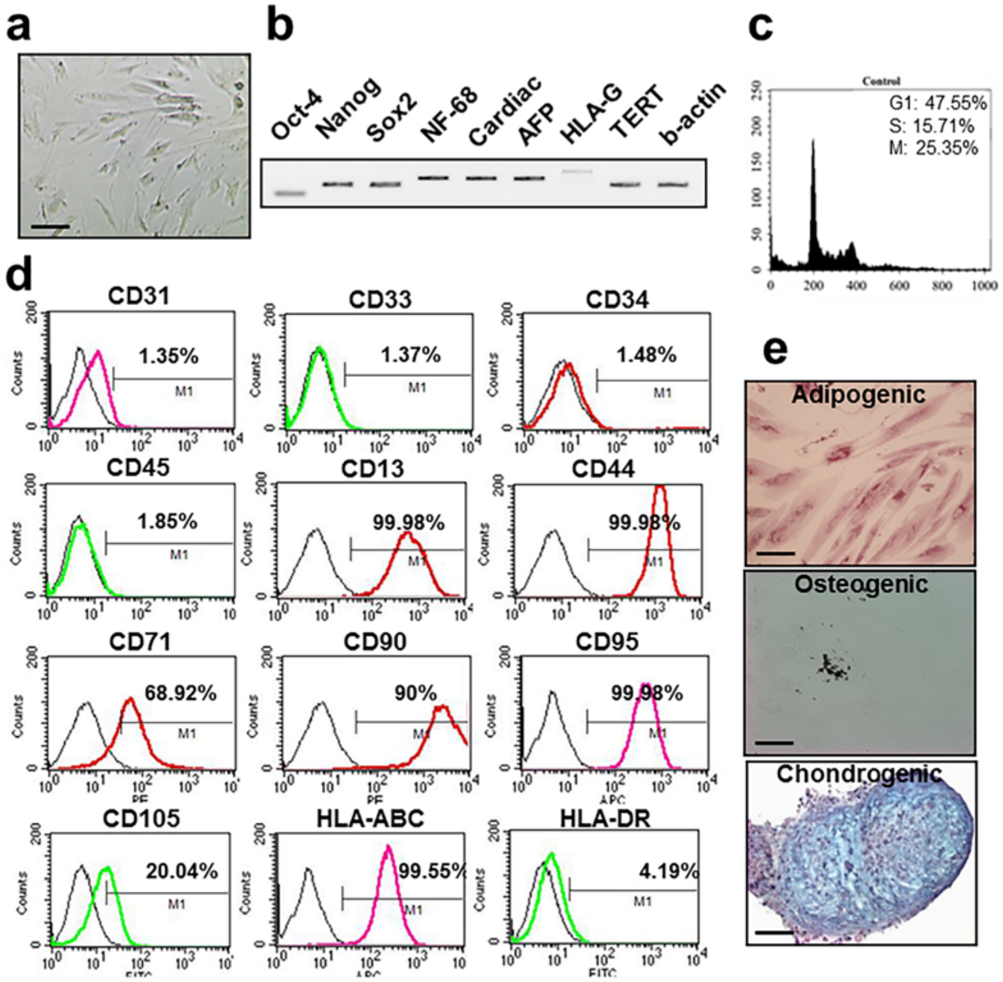

Supplementary Fig. 1. Characterization of PD-MSCs.

(a) The shape of the PD-MSCs resembled that mesenchymal stem cells derived from bone marrow or adipose tissues (x100). (b) The mRNA expression levels of stem cell markers in PD-MSCs. (c) FACS-based cell cycle analysis of PD-MSCs. (d) The expression of surface markers in PD-MSCs, as assessed using FACS. The percentage is indicated with the fluorescence. (e) Differentiation of PD-MSCs. Adipogenic, osteogenic and chondrogenic differentiation were analyzed by Oil Red O, von Kossa and Alcian blue staining, respectively. Scale bars: 100 μm.

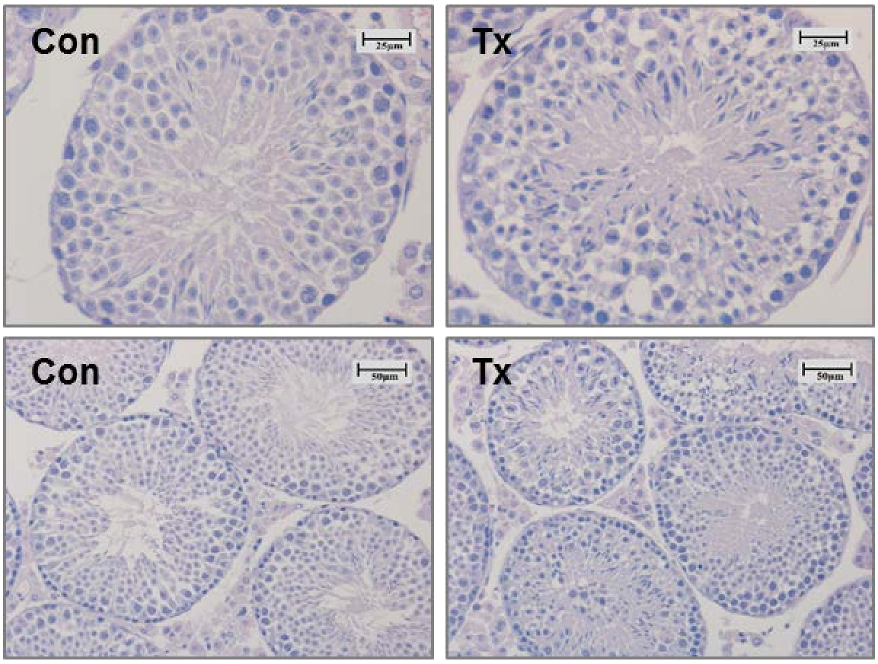

Supplementary Fig. 2. Teratoma formation in NOD/SCID mice after PD-MSCs transplantation. Histopathological analysis of the normal NOD/SCID mice testis and PD-MSCs transplanted NOD/SCID mice testis until 14 weeks using H&E. Con: control, Tx: PD-MSCs transplanted group. Scale bars: 25 μm (upper), 50 μm (lower).

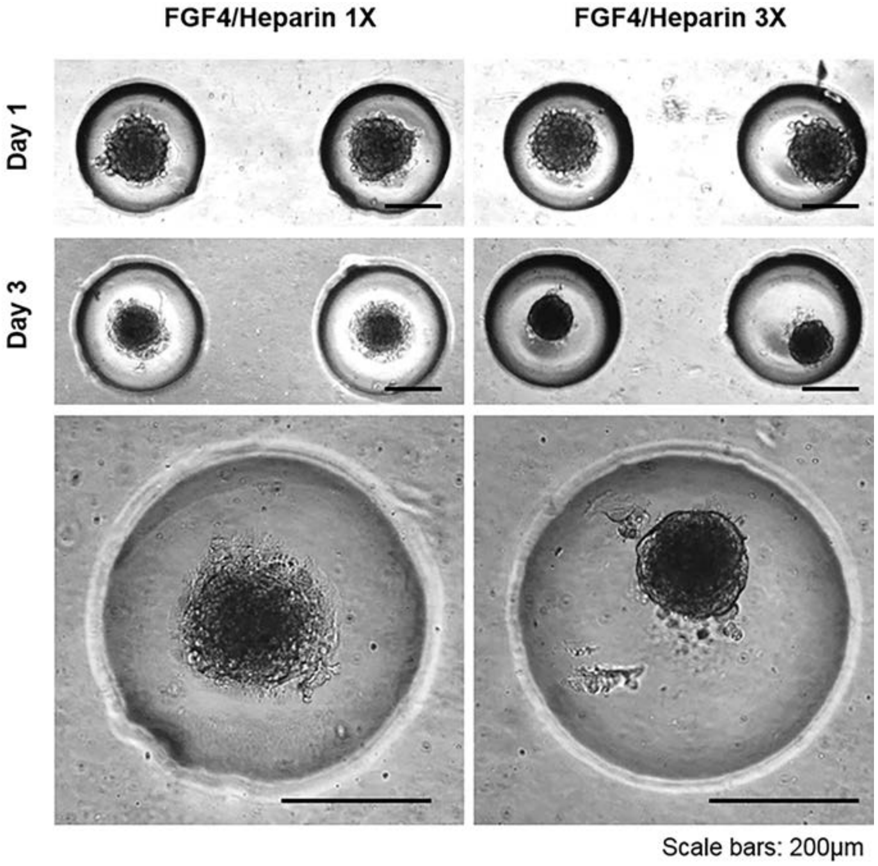

Supplementary Fig. 3. Optimization of the medium/supplements for culture of PD-MSC spheroids.
(a) PD-MSC spheroids show attachment onto the PDMS substrate, showing differentiated state when cultured in medium containing 25 ng/ml FGF-4 and 1 μg/ml heparin. (b) PD-MSC spheroids cultured in medium containing doses of FGF-4 and heparin 3-fold higher than those used in (a) do not appear differentiated, and show clear spheroidal structure.
